# Effects of 1α,25-Dihydroxyvitamin D_3_ on the Pharmacokinetics of Procainamide and Its Metabolite N-Acetylprocainamide, Organic Cation Transporter Substrates, in Rats with PBPK Modeling Approach

**DOI:** 10.3390/pharmaceutics13081133

**Published:** 2021-07-25

**Authors:** Anusha Balla, Yoo-Seong Jeong, Hyo-Jung Kim, Yun-Jong Lee, Suk-Jae Chung, Yoon-Jee Chae, Han-Joo Maeng

**Affiliations:** 1 College of Pharmacy, Gachon University, Incheon 21936, Korea; aanushaballa@gmail.com; 2 Research Institute of Pharmaceutical Sciences, College of Pharmacy, Seoul National University, Seoul 08826, Korea; jus2401@snu.ac.kr (Y.-S.J.); sukjae@snu.ac.kr (S.-J.C.); 3 Department of Pharmacology, Sungkyunkwan University School of Medicine, Suwon 16419, Korea; hjung93@skku.edu (H.-J.K.); ylee69@skku.edu (Y.-J.L.); 4 College of Pharmacy, Woosuk University, Wanju-gun 55338, Korea

**Keywords:** 1α,25-dihydroxyvitamin D_3_, rOCTs, rMATE1, procainamide, N-acetylprocainamide, PBPK modeling

## Abstract

In this study, possible changes in the expression of rat organic cationic transporters (rOCTs) and rat multidrug and toxin extrusion proteins (rMATEs) following treatment with 1α,25-dihydroxyvitamin D_3_ (1,25(OH)_2_D_3_) were investigated. Rats received intraperitoneal administrations of 1,25(OH)_2_D_3_ for four consecutive days, and the tissues of interest were collected. The mRNA expression of rOCT1 in the kidneys was significantly increased in 1,25(OH)_2_D_3_-treated rats compared with the control rats, while the mRNA expressions of rOCT2 and rMATE1 in the kidneys, rOCT1 and N-acetyltransferase-II (NAT-II) in the liver, and rOCT3 in the heart were significantly decreased. Changes in the protein expression of hepatic rOCT1 and renal rOCT2 and rMATE1 were confirmed by western blot analysis. We further evaluated the pharmacokinetics of procainamide (PA) hydrochloride and its major metabolite N-acetyl procainamide (NAPA) in the presence of 1,25(OH)_2_D_3_. When PA hydrochloride was administered intravenously at a dose 10 mg/kg to 1,25(OH)_2_D_3_-treated rats, a significant decrease in renal and/or non-renal clearance of PA and NAPA was observed. A physiological model for the pharmacokinetics of PA and NAPA in rats was useful for linking changes in the transcriptional and translational expressions of rOCTs and rMATE1 transporters to the altered pharmacokinetics of the drugs.

## 1. Introduction

Drug transporters are crucial factors that affect the pharmacokinetics of therapeutic drugs. Changes in the expression and/or function of such biological proteins may alter drug disposition, toxicology, and pharmacological responses at the site of action. Previous studies [1,2] have reported changes in the expression of adenosine triphosphate (ATP)-binding cassette or solute carrier membrane transporters in various tissues, including in the kidney, liver, and brain, under pathological conditions. The administration of some therapeutic drugs has also been reported to alter the expression levels of transporters [3,4]. The gene expression of transporters and drug-metabolizing enzymes, which may affect the pharmacokinetic profiles of drugs, is known to be regulated by nuclear receptor proteins including pregnane X receptor (PXR), constitutive androstane receptor (CAR), farnesoid X receptor (FXR), and vitamin D receptor (VDR) [5,6].

Among these nuclear receptor proteins, VDR is an intracellular hormone receptor, which exerts its biological effects by stimulating downstream signaling involved in various physiological activities such as calcium homeostasis, bone mineralization, and cell differentiation [7,8]. In addition, VDR regulates cytochrome P450 (CYP) and the transporters expressed in various tissues, as described above. For example, VDR has been reported to regulate CYP3A4 expression in the human intestine [9] and CYP3A4, CYP2B6, and CYP2C9 in human hepatocytes [10] in the presence of 1α,25-dihydroxyvitamin D_3_ (1,25(OH)_2_D_3_), a natural ligand of VDR, which may thus affect the first-pass drug metabolism and systemic pharmacokinetic profiles. In addition, intestinal multidrug resistance protein 1 (MDR1) expression was induced by 1,25(OH)_2_D_3_ via the binding of VDR and retinoid X receptor α (VDR/RXRα) to several VDREs (i.e., vitamin D-response element) [11]. The treatment of rats with 1,25(OH)_2_D_3_ reduced renal mRNA levels of rat oligopeptide transporter 1 (rPEPT1) and rat organic anion transporters 1 and 3 (rOAT1 and rOAT3), resulting in a significant decrease in the renal clearance of cefdinir and cefadroxil [12,13]. Although these observations support the importance of VDR in the molecular regulation of several metabolizing enzymes/transporters and their potential impact on the pharmacokinetics of therapeutic agents, information on the effect of 1,25(OH)_2_D_3_ on the expression of other clinically recognized transporters, such as organic cation transporters (OCTs) or multidrug and toxin extrusion proteins (MATEs), remains lacking.

Procainamide (PA), which is classified as a type IA antiarrhythmic drug, is commonly used for the treatment of ventricular arrhythmias and stable sustained monomorphic ventricular tachycardia [14,15]. PA exists in a cationic form in biological matrices (i.e., with a basic pKa value of 9.04) and is a substrate of hOCTs [16,17] and hMATE1 [18]. The elimination of PA occurs via renal excretion as well as hepatic metabolism by N-acetyltransferase-II (NAT-II) to form N-acetylprocainamide (NAPA), which is also an anti-arrhythmic agent [19,20,21]. Using a pharmacokinetic modeling approach, the findings of a previous study [22] suggested that the functional variation of renal transporters such as OCT2 and MATE1 may affect the pharmacokinetics of PA and NAPA. Therefore, we hypothesized that functional changes in transporters, potentially due to VDR regulation, in the presence of 1,25(OH)_2_D_3_ may impact on the pharmacokinetics of PA and NAPA. Because of the narrow therapeutic indices of PA and NAPA [23], studies on the variable expression of cationic transporters by VDR are needed.

Therefore, the primary objective of this study was to investigate the effects of 1,25(OH)_2_D_3_ on the expression levels of rOCTs in various tissues and of rMATE1 in the kidneys. To further examine whether the observed changes in transporter expression influenced the pharmacokinetics of PA and NAPA, the systemic pharmacokinetics, urinary excretion, and tissue distribution of both drugs were studied in rats with and without 1,25(OH)_2_D_3_ treatment. Using a physiologically-based pharmacokinetic (PBPK) model established for the pharmacokinetics of PA and NAPA in rats [22], the altered pharmacokinetics of the drugs were quantitatively assessed in this study.

## 2. Materials and Methods

### 2.1. Materials

In this study, 1,25(OH)_2_D_3_, procainamide hydrochloride (PA HCl), N-acetylprocainamide (NAPA), and N-propionylprocainamide (i.e., internal standard (IS) for ultra-high performance liquid chromatography (UHPLC) analysis), acetic acid, triethylamine, and corn oil were purchased from Sigma-Aldrich (St. Louis, MO, USA). Water was purified in-house using an aquaMAX™ ultra-pure water purification system (YL Instruments, Anyang, Korea). Methanol was purchased from Honeywell Burdick and Jackson (Ulsan, Korea). All other chemicals and solvents were of reagent or HPLC grade and used without further purification.

### 2.2. Experimental Animals and Treatment of Rats with 1,25(OH)_2_D_3_

The animal experiments were performed in accordance with the Guidelines for Animal Care and Use of Gachon University (approval number: GIACUC-R2017011, approval date: 25 May 2017). Sprague-Dawley (SD) rats (7–8-weeks old, 260–320 g) were purchased from Nara Bio Technology, Seoul, Korea. Rats were acclimatized for 1 week with free access to food and water. Animals were maintained under a 12:12 h light–dark cycle in accordance with the animal protocols.

To investigate the effect of 1,25(OH)_2_D_3_ treatment on the expression of transporters in rat tissues, SD rats were treated in the following manner, as previously reported [12,13,24,25]. Rats in the control and treatment groups intraperitoneally received a solution of 0.0452% ethanol in corn oil filtered by a syringe filter (Sartorius, Goettingen, Germany) without or with 1,25(OH)_2_D_3_ (at a concentration of 2.56 µM) for four consecutive days at the same time of each day (9:30–10:00 a.m.). The rats were weighed daily during the treatment period. On the 5th day (i.e., 24 h after the last dose), the control and 1,25(OH)_2_D_3_-treated rats were anesthetized with an intraperitoneal injection of tiletamine HCl/zolazepam HCl (20 mg/kg, Zoletil 50^®^; Virbac Laboratories, Carros, France) and xylazine HCl (10 mg/kg, Rompun^®^; Bayer AG, Leverkusen, Germany), which were then used in subsequent studies.

### 2.3. Effect of 1,25(OH)_2_D_3_ on the Gene Expression of rOCTs, rMATE1, and rNAT-II in Rat Tissues

To examine whether 1,25(OH)_2_D_3_ treatment affects the expression of various genes in rat tissues, heart, liver, and kidney samples were collected from anesthetized rats and immediately frozen in liquid nitrogen. The expression levels of rOCT3 in heart, rOCT1, rOCT2, rOCT3, and rMATE1 in kidney, and rOCT1 and NAT-II (i.e., metabolic converting enzyme of PA into NAPA) in liver were determined by real-time quantitative polymerase chain reaction (qPCR) and normalized to that of rat glyceraldehyde 3-phosphate dehydrogenase (rGAPDH), as previously reported [13,26]. Briefly, RNAiso Plus (Takara Bio Inc., Shiga, Japan) was used to extract total RNA from 100 mg tissue samples according to the manufacturer’s protocol. The purity and concentration of total RNA were measured using a NanoDrop 2000c spectrophotometer (Thermo Scientific, Lenexa, KS, USA). A first-strand cDNA kit (Takara Bio Inc., Shiga, Japan) was used to synthesize cDNA from approximately 1 µg of total RNA. The synthesized cDNA was subjected to qPCR assays using SYBR^®^ Premix Ex Taq™ (Takara Bio Inc., Shiga, Japan) on a Stratagene Mx3005P system (Agilent Technologies, Cedar Creek, TX, USA). The qPCR primer pairs used were as follows: rGAPDH forward primer 5′-CGCTGGTGCTGAGTATGTCG-3′; rGAPDH reverse primer 5′-CTGTGGTCATGAGCCCTTCC-3′; rOCT1 forward primer 5′-TTTAACCTGGTGTGTGGAGACG-3′; rOCT1 reverse primer 5′-AGGAAGAAGCCCAAGTTCACAC-3′; rOCT2 forward primer 5′-CGGTGCTATGATGATTGGCTAC-3′; rOCT2 reverse primer 5′-CCAGGCATAGTTGGGAGAAATC-3′; rOCT3 forward primer 5′-ATATCCTGTTTCGGCGTTGG-3′; rOCT3 reverse primer 5′-TTTCCAAACACCCCTTGCAG-3′; rMATE1 forward primer 5′-CTCTTCATCAACACCGAGCA-3′; rMATE1 reverse primer 5′-ACCCATCACCCCAAGATGTA-3′; rNAT-II forward primer 5′-GCGAGAAGTGGTCCTGAGTAG-3′; rNAT-II reverse primer 5′-CAAAGGGAATAGCCCGTATCT-3′ [27,28,29,30]. Amplification and detection were performed according to the manufacturer’s protocol, using the MxPro-Mx3005P system (Agilent Technologies, Santa Clara, CA, USA) at 95 °C for 10 min, with 40 cycles of 95 °C for 15 s and 55 °C for 30 s, followed by dissociation curve analysis. The fold expression was represented as 2^−(ΔΔCT)^ to quantify relative mRNA expression [12].

### 2.4. Effect of 1,25(OH)_2_D_3_ on the Protein Expression of rOCTs and rMATE1 in Rat Liver and Kidney

In this study, the protein expression levels of rOCT1, rOCT2, and rMATE1 in kidney and rOCT1 in liver were determined by western blotting. For protein extraction, RIPA buffer (#89900, Thermo Fisher Scientific, Waltham, MA, USA) was used for homogenization of rat liver or kidney tissues with protease/phosphatase inhibitors using a Diax 900 homogenizer. The homogenized liver or kidney lysates were incubated in ice for 30 min (for complete lysis) by vortexing every 5 min, followed by centrifugation at 14,000 × *g* for 30 min. The supernatant proteins were collected and diluted by 10-fold, and then the protein concentrations were determined using BCA Protein Assay Kit (#23227, Thermo Fisher Scientific, Waltham, MA, USA). Bovine serum albumin (BSA) was applied for standards. Lysates were mixed with 2× Laemmli buffer (Bio-Rad, Hercules, CA, USA) supplemented with β-mercaptoethanol (Bio-Rad) and boiled for 5 min. Proteins were separated by sodium dodecyl sulfate-polyacrylamide gel electrophoresis (SDS-PAGE) and transferred onto nitrocellulose membranes (0.45 µm, Bio-Rad; cat##162-0115) for immunoblotting. Immunoblotting was accomplished with the specific antibodies, and the bands were visualized via chemiluminescence (#34577, Thermo Fisher Scientific, Waltham, MA, USA). The primary antibodies were used as follows: rabbit SLC47A1 (MATE1) antibody (#ANT-131, Alomone Labs, Jerusalem, Israel), rabbit OCT2 antibody (#OCT21-A, ALPHA DIAGNOSTIC, San Antonio, TX, USA), rabbit OCT1 antibody (#OCT11-A, ALPHA DIAGNOSTIC, TX, USA), and HRP-conjugated β-actin mouse antibody (Sigma; cat#A3854; 1:10,000). In addition, the following secondary antibodies were used: HRP-conjugated goat anti-rabbit IgG antibody (Genetex; cat# GTX-213110-01; 1:5000). Densitometric analysis of the bands was performed using ImageJ software (NIH; rsb.info.nih.gov/ij, assessed on 4 March 2021).

### 2.5. Effect of 1,25(OH)_2_D_3_ on the Pharmacokinetics of PA and NAPA in Rats

On the 5th day after vehicle treatment with or without 1,25(OH)_2_D_3_, rats were anesthetized, and the femoral vein (for administrating drugs and replenishing body fluids) and artery (for collecting blood samples) were cannulated with polyethylene tubing (PE50; Clay Adams, Parsippany, NJ, USA). After recovery from anesthesia, 10 mg/mL PA HCl dissolved in normal saline (HK inno.N Corp., Seoul, Korea) was administered intravenously at a dose of 10 mg/kg to both the control and treatment group (*n* = 9 rats each). Blood was then collected at 0 (blank), 1, 5, 15, 30, 60, 120, 180, 240, 360, and 480 min after drug administration. The plasma fraction was separated by centrifugation of blood samples for 15 min at 14,000 rpm at 4 °C and then stored at −20 °C until subsequent analysis. To quantify PA and NAPA in the plasma, 200 μL of internal standard (IS) solution (200 ng/mL in methanol) was added to an aliquot of 100 μL of plasma and then vortexed for 1 min. The mixture was centrifuged for 15 min at 14,000 rpm and 4 °C, and 2 µL of the supernatant was then injected into the UHPLC system (see below).

### 2.6. Effects of 1,25(OH)_2_D_3_ on the Urinary and Fecal Excretion of PA and NAPA in Rats

To determine the effect of 1,25(OH)_2_D_3_ on the urinary and fecal excretion of PA and NAPA, rats were administered 10 mg/kg PA HCl in normal saline (1 mL/kg) via the tail vein (*n* = 7 for each group) on the 5th day of the treatment, and then placed in individual metabolic cages. Urine and feces were collected separately using a urine-feces separator. Water was freely available to the rats, while food was given 8 h after the administration of PA HCl. Urine samples were collected at intervals of 0–2, 2–4, 4–6, 6–8, and 8–24 h after drug administration. The samples obtained during 0–8 h were weighed and diluted 100-fold with distilled deionized water (DDW), while those obtained at 8–24 h were centrifuged for 30 min to separate food particles at 300 rpm and 4 °C, weighed, and then diluted 50-fold with DDW. When necessary to analyze urine samples, an analytical method was used as previously described, with slight modification [31]. A 90 μL volume of urine samples was spiked with 10 μL of IS solution (2 µg/mL) followed by the addition of 40 µL of 4 N NaOH. For the extraction of PA and NAPA, the resulting samples were added to 800 µL of methylene chloride, and the mixture was vortexed for 1 min and centrifuged for 10 min at 10,000 × *g* and 4 °C. The organic extracts (from the bottom layer) obtained by a series of liquid–liquid extraction processes were reconstituted with 100 µL methanol. The samples were centrifuged at 14,000 rpm for 15 min at 4 °C, and then 2 µL of the supernatant was injected into the UHPLC system (see below).

Feces were collected at intervals of 0–24 h and 24–48 h and added to an adequate volume of 0.9% NaCl solution. Samples were homogenized to obtain a fecal slurry, which was then centrifuged for 20 min at 3000 rpm and 4 °C. The supernatant was diluted five-fold with DDW and stored at −80 °C until analysis.

### 2.7. Effects of 1,25(OH)_2_D_3_ on the Tissue Distribution of PA and NAPA at Steady State

To evaluate the effects of 1,25(OH)_2_D_3_ treatment on the tissue distribution of PA and NAPA, the tissue-to-plasma partition coefficient at steady state ($K_{p,ss}$) was determined for various tissues. As described above, the rats were anesthetized on the 5th day of treatment, and the femoral vein and artery were cannulated. Following recovery from anesthesia, PA HCl was injected at a loading dose of 1.4 or 1.5 mg/kg (for the control or treatment group, respectively) dissolved in normal saline, followed by constant infusion of the drug at a rate of 2.5 mg/kg/h (i.e., as a maintenance dose of 0.784 mg/rat for control and 0.640 mg/rat for 1,25(OH)_2_D_3_-treated rats) (*n* = 5 for each group), using a syringe pump (model no. NE-1800, New Era Pump System Inc., Farmingdale, NY, USA). Blood samples were obtained at 1, 5, 15, 30, 45, and 60 min after the initiation of PA administration via the femoral artery. Based on no significant difference among plasma concentrations of the drug at 30, 45, and 60 min from using one-way analysis of variance (ANOVA), the steady state condition was confirmed at 60 min. After 60 min of PA administration, therefore, the rats were rapidly sacrificed, and six major tissues (i.e., brain, heart, kidney, liver, lung, and spleen) were collected. After the wet weights of tissue samples were measured, a two-fold volume of PBS was added to homogenize brain and spleen, whereas a five-fold volume of PBS was added for heart, kidney, liver, and lung. It was assumed that the densities of all tissue samples are consistent in this study. The tissue homogenates were kept at −80 °C until analysis. $K_{p,ss}$ was calculated by dividing the tissue concentration of each drug by its plasma concentration at 60 min.

### 2.8. Effects of 1,25(OH)_2_D_3_ on the In Vitro Metabolic Conversion of PA into NAPA in Rat Liver S9 Fractions

In this study, rat liver S9 fractions were prepared according to a previously described method, with slight modifications [32]. Briefly, rats were anesthetized on the 5th day of the treatment schedule, and the liver was collected and immediately frozen by immersion in liquid nitrogen. Liver samples were homogenized in a 2.5-fold volume of buffer consisting of 0.154 M potassium chloride and 50 mM tris-hydrochloride in 1 mM ethylenediaminetetraacetic acid (EDTA) adjusted to pH 7.4, using a Wheaton™ Dounce tissue grinder. The resultant homogenate was centrifuged at 9000 × *g* for 20 min at 4 °C to obtain the liver S9 fraction (i.e., the supernatant). The protein concentration in the fractions was determined by Lowry’s method using Lowry reagent (Sigma-Aldrich Co., St Louis, MO, USA), according to the manufacturer’s protocol. The rat liver S9 fraction obtained was stored at −80 °C until analysis.

For in vitro metabolism study, a reaction mixture of liver S9 fractions containing PA was prepared at a concentration of 2 mg protein/mL in 100 mM potassium phosphate buffer adjusted to pH 7.4, at a total volume of 500 µL. The final concentrations of PA in the solutions were 50, 500, and 5000 µM. After the mixture was preincubated at 37 °C for 5 min in a Benchmark Multi-Therm Shaking Vortexer set at 200 oscillations/min, the reaction was initiated by adding 10 µL of acetyl-CoA (at a final concentration of 2 mM) and then vortexing. An aliquot (50 µL) was aspirated from the mixture at 0, 15, and 30 min after initiation, and the reaction was terminated by adding 100 µL of ice-cold methanol containing IS (200 ng/mL). The samples were vortexed and centrifuged at 12,000 × *g* for 10 min at 4 °C. The supernatant was injected into the UHPLC system to determine the concentration of NAPA (see below).

### 2.9. Determination of the Free Fraction of PA in Plasma and Incubation Mixture of Rat Liver S9 Fractions

In the present study, the binding of PA in the plasma and liver S9 fraction mixture obtained from the control and 1,25(OH)_2_D_3_ treated rats was examined using Amicon^®^ Ultra-3 K centrifugal filter units (Merck Millipore, Ltd., Tullagreen, Carrigtwohill, Ireland). Plasma was obtained from control and 1,25(OH)_2_D_3_-treated rats (*n* = 3 each), and the rat liver S9 fraction was prepared as described above (*n* = 5 each). Twenty-microliters of PA HCl stock solution was spiked into 980 µL of the plasma or S9 fraction mixture, resulting in a final concentration of 5 µg/mL or 50 µM, respectively. After 100 µL of the aliquot was aspirated ($C_{1}$) as a plasma standard to calculate $C_{2}$ concentration, the remaining solution was incubated at 37 °C for 12 min. Then, 500 µL of the incubated sample was transferred to an Amicon^®^ Ultra centrifugal filter unit, and 100 µL was aspirated ($C_{2}$) from the transferred solution. The centrifugal filter units were centrifuged at 37 °C and 5000 rpm for 12 min. The filtrate was weighed ($C_{f}$), and 100 µL of the sample remaining in the upper part of the centrifugal unit was collected ($C_{3}$). All obtained samples ($C_{1}$, $C_{2}$, and $C_{3}$) were each mixed with 200 µL of methanol containing 200 ng/mL IS, while the weighed filtrate ($C_{f}$) was added to a two-fold volume of the IS solution. The mixture was vortexed for 1 min and centrifuged for 15 min at 14,000 rpm and 4 °C. The supernatant was then transferred to vials for UHPLC analysis.

To determine non-specific binding in the preparation of ultrafiltrate samples, 20 µL of PA HCl stock solution (250 µg/mL) was added to 980 µL of PBS. Similarly, 500 µL of the mixture was transferred into centrifugal filter units and 100 µL was sampled from the transferred mixture ($C_{Before}$). The centrifugal filter units were then centrifuged for 6 min at 37 °C and 5000 rpm, and the filtrate was weighed ($C_{After}$). Using IS solution in methanol (200 ng/mL), the resulting samples were vortexed for 1 min and centrifuged for 15 min at 4 °C and 14,000 rpm min, as described above, and injected into UHPLC. The free fraction was calculated as follows:

(1) $$\mathrm{Non}-\mathrm{specific}\;\mathrm{binding}\;\left(\mathrm{NSB}\right)\%=\frac{\left(C_{Before\;}-\;C_{After}\right)}{C_{Before}}\;\times \;100$$

(2) $$\mathrm{Free}\;\mathrm{fraction}\%=\frac{C_{rf}}{C_{2}}\;\times \;100$$

(3) $$\mathrm{Corrected}\;\mathrm{filtrate}\;\mathrm{concentration}\;(C_{rf})=\frac{\left[\mathrm{Measured}\;\mathrm{filtrate}\;\mathrm{concentration}\;\left(C_{f}\right)\right]}{100\;-\;\mathrm{NSB}}\;\times \;100$$

(4) $$\mathrm{Recovery}\%=\frac{\left[C_{f}\;\times \;\mathrm{filtrate}\;\mathrm{wt}.+C_{3}\;\times \;\left(0.4\;-\;\mathrm{filtrate}\;\mathrm{wt}.\right)\right]}{C_{2}\;\times \;0.4}\;\times \;100$$

### 2.10. UHPLC Analysis

UHPLC analysis was performed using an Agilent Technologies 1290 Infinity II UHPLC system equipped with a multisampler (G7167B), a flexible pump (G7104A), a multicolumn thermostat (MCT) (G7116B), and a diode array detector (DAD) detector (G7117A). A Synergi polar-RP column 80A (150 × 2.0 mm, 4 µm; Phenomenex, Torrnce, CA, USA) column was used for analysis. The mobile phase was composed of 1% acetic acid (pH 5.5) and methanol (76:24, *v/v*) and eluted in isocratic mode at a flow rate of 0.2 mL/min. The injection volume was 2 µL, and the detection wavelength was 280 nm. The column and autosampler trays were maintained at 25 and 4 °C, respectively.

### 2.11. Application of Physiologically-Based Pharmacokinetic Modeling for PA and NAPA in the Absence and Presence of 1,25(OH)_2_D_3_ Treatment in Rats

PA has previously [18,33] been reported to be a substrate of OCT and MATE transporters, which are thus considered to play crucial roles in the disposition of PA [34,35,36]. In this study, mRNA and protein expression levels of renal transporters, including rOCT2 and rMATE1, were found to be reduced in the presence of 1,25(OH)_2_D_3_ treatment. In addition, mRNA and protein expression levels of rOCT1 in rat kidney increased following 1,25(OH)_2_D_3_ treatment (see Section 3). Since the pharmacokinetics of PA and NAPA in rats was also changed following 1,25(OH)_2_D_3_ treatment, we reasoned that the relevance of the observed change may be mechanistically addressed by applying a physiological model for the pharmacokinetics of PA and NAPA. In the literature [22], a PBPK model integrating the active transport kinetics in a semi-mechanistic kidney model was proposed to predict drug–drug interactions of PA and NAPA with cimetidine in rats. Therefore, we utilized the same model structure to predict the pharmacokinetic changes in PA and NAPA following 1,25(OH)_2_D_3_ treatment, along with slightly modified parameter values.

The parameters necessary for PBPK calculations, in accordance with the previous model [22], were applied (see Section 3). Briefly, while the systemic pharmacokinetic profiles of PA and NAPA in the control group were consistent with those reported previously, the cumulative urinary recovery was somewhat affected following treatment with vehicle for 4 consecutive days (i.e., 0.0452% ethanol in filtered corn oil; 1 mL/kg) with a factor of 1.29 and 0.836 for PA and NAPA, respectively. In addition, the $K_{p,ss}$ values in a few tissues differed from those reported previously (e.g., brain and lung for PA, and brain, heart, and liver for NAPA; greater than a factor of two). Therefore, an approach similar to that previously used to calculate PBPK model parameters was considered, utilizing the currently observed $K_{p,ss}$ values in our PBPK model.

Using a semi-mechanistic kidney model [37], the clearances of basolateral uptake ($PS_{in}$) and apical efflux ($CL_{u,int,r}$) of PA and NAPA were incorporated. Similarly, $PS_{in}$ was assumed to consist of active and passive drug uptake (i.e., $PS_{act}$ and $PS_{pas}$). Despite the relatively lower expression of rOCT1 compared to rOCT2 in rat kidneys (38.3 compared to 254 pmol/g kidney) [38], $PS_{act}$ was assumed to be composed of $PS_{rOCT1}$ and $PS_{rOCT2}$ since this study revealed a significant increase in rOCT1 expression along with a significant decrease in rOCT2 by 1,25(OH)_2_D_3_ treatment. Interestingly, there has been some evidence of a marked difference in the affinity of PA between rOCT1 (e.g., IC50 values for representative substrate uptake, ranging from 3.56 to 12 µM) and rOCT2 (from 90 to 748 µM) [39,40,41]. However, the estimation of $PS_{rOCT1}$ and $PS_{rOCT2}$ could not be separated because of the absence of information on the flipping rate ($k_{cat}$) of PA or NAPA by each transporter. To apply the PBPK model in the presence of 1,25(OH)_2_D_3_ treatment, therefore, we estimated the fold change in the overall active uptake ($R_{act}=PS_{act,vitD}/PS_{act}$; Equation (A14), see Appendix A), which was assumed to be consistent for PA and NAPA, while the level of rMATE1 protein expression observed in this study was used for the functional change of the proteins. Detailed description of differential equations used for the current modeling analysis are shown in Appendix A.

### 2.12. Statistical Analysis

As an evaluation of the predictability of PBPK model for PA and NAPA, the absolute average fold error (AAFE) was calculated for the comparison of model-predicted concentrations/amounts with the observed values as follows:

(5) $$AAFE=10^{\frac{1}{n}\sum^{}\left|log\frac{C_{pred}}{C_{obs}}\right|}$$

where $C_{pred}$ and $C_{obs}$ refer to the predicted and observed concentrations, respectively, and n indicates the number of observations.

Pharmacokinetic parameters were calculated by non-compartmental analysis (NCA) using WinNonlin software (Version 5.0.1., Pharsight Corporation, Mountain View, CA, USA). Renal clearance ($CL_{R}$) was calculated by dividing the amount of drug excreted in the urine by the area under the plasma concentration-time curve from time 0 to infinity ($AUC_{inf}$). Non-renal clearance ($CL_{NR}$) was obtained by subtracting $CL_{R}$ from total clearance (CL). $R_{act}$ was fitted to the PBPK model using ADAPT 5 with the variance model as follows [42]:

(6) $$V_{i}={\left(\sigma_{1}+\sigma_{2}\cdot Y_{i}\right)}^{2}$$

where, $V_{i}$ is the variance of the *i*th data point, $Y_{i}$ is the *i*th model prediction, and $\sigma_{1}$ and $\sigma_{2}$ are variance model parameters. The ADAPT model code used for estimating $R_{act}$ is provided in the Supplementary Material. When the numerical integration is necessary, computations were conducted using the fourth-order Runge–Kutta method built in Berkeley Madonna software (version 8.3.18; University of California, Berkeley, CA, USA).

Differences between two groups were analyzed using a two-tailed Student’s *t*-test. In this study, data are expressed as the mean ± standard deviation (S.D.), and *p* values less than 0.05 were regarded as statistically significant.

## 3. Results

### 3.1. Effects of 1,25(OH)_2_D_3_ on the mRNA/Protein Expression of OCT and MATE Transporters in Rats

**Figure 1:**
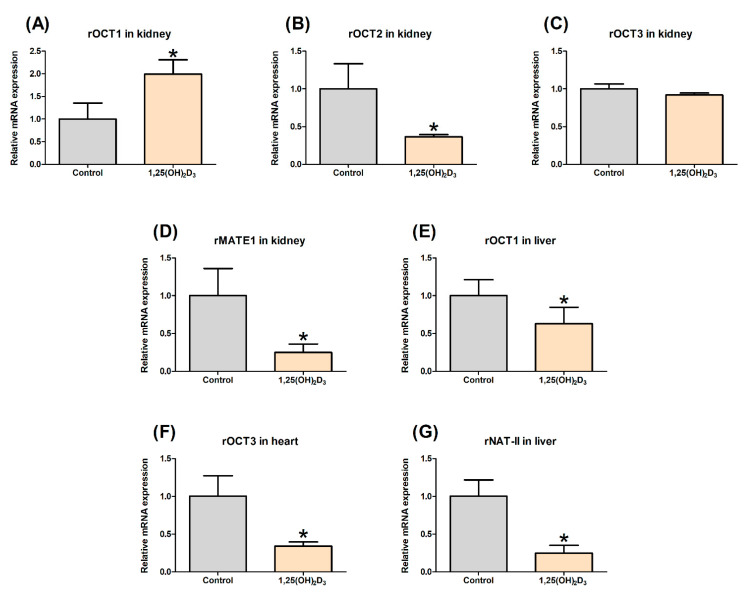
Effects of 1,25(OH)_2_D_3_ on the mRNA expression levels of (**A**) renal rOCT1, (**B**) renal rOCT2, (**C**) renal rOCT3, (**D**) renal rMATE1, (**E**) hepatic rOCT1, (**F**) rOCT3 in heart, and (**G**) rNAT-II in liver tissue from control (gray color) and 1,25(OH)_2_D_3_-treated rats (light orange color). * Indicates *p* < 0.05.

**Figure 2:**
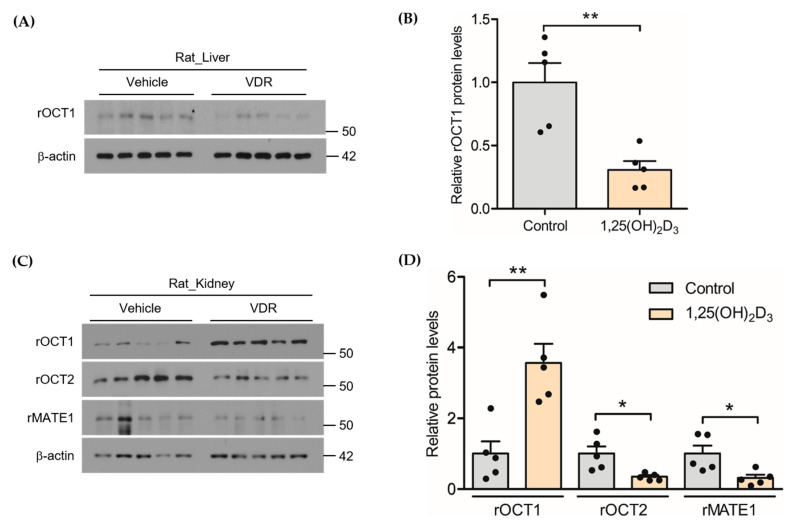
Effects of 1,25(OH)_2_D_3_ on the protein expression levels of hepatic rOCT1 and renal rOCT1, rOCT2, and rMATE1. (**A**) Western blotting for hepatic rOCT1, (**B**) densitometry of western blotting for hepatic rOCT1 (**C**) Western blotting for renal rOCT1, rOCT2, and rMATE1, (**D**) densitometry of western blotting for renal rOCT1, rOCT2, and rMATE1 in control and 1,25(OH)_2_D_3-_treated groups (*n* = 5 each group). ** Indicates *p* < 0.01 and * indicates *p* < 0.05.

The primary objective of this study was to investigate the potential effect of 1,25(OH)_2_D_3_ on the expression of cationic transporters in various tissues. Figure 1 shows the changes in the mRNA expression of the OCT and MATE transporters and the rNAT-II enzyme in various tissues from the control and 1,25(OH)_2_D_3_-treated rats. qPCR analysis revealed that the mRNA expression of rOCT1 was significantly increased in kidney tissue from 1,25(OH)_2_D_3_-treated rats compared with that from control rats (i.e., 1.98-fold of control), whereas the mRNA levels of rOCT2 and rMATE1 were significantly decreased (i.e., 0.366- and 0.248-fold of control, respectively). No change in the mRNA expression of rOCT3 in the kidneys was observed between the two groups (0.921-fold). In addition, the mRNA expressions of heart rOCT3 and hepatic rOCT1 and rNAT-II enzymes were significantly lower than that in the control following treatment with 1,25(OH)_2_D_3_, with 0.342, 0.688, and 0.248-fold of the control values, respectively. Moreover, as shown in Figure 2, a western blot analysis confirmed that the rOCT1 protein levels were significantly reduced in the liver (0.31-fold of control, *p* < 0.01, Figure 2B) and that the rOCT2 (0.35-fold of control) and rMATE1 (0.31-fold of control) levels were significantly reduced in the kidneys (*p* < 0.05, Figure 2D) by 1,25(OH)_2_D_3_ treatment, whereas the rOCT1 expression in the kidneys was induced by treatment with 1,25(OH)_2_D_3_ (*p* < 0.01, Figure 2D). Collectively, these results indicate that the mRNA/protein expression levels of cationic transporters and enzymes were altered by 1,25(OH)_2_D_3_ treatment in rats.

### 3.2. Effects of 1,25(OH)_2_D_3_ on the Pharmacokinetics of PA and NAPA Following the Intravenous Administration of PA HCl to Rats

**Figure 3:**
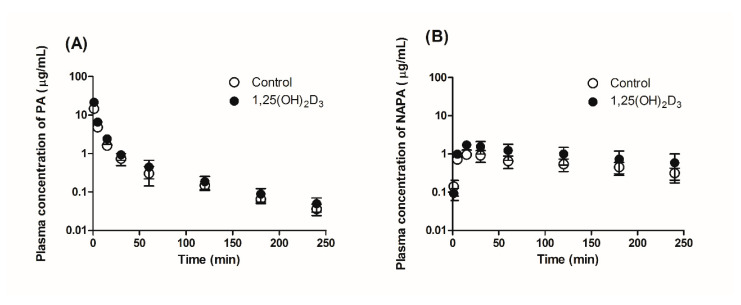
Plasma concentration-time profiles of (**A**) PA and (**B**) NAPA following intravenous administration of 10 mg/kg PA HCl to control (open circles) and 1,25(OH)_2_D_3_-treated rats (closed circles) (*n* = 9 per group).

Following the intravenous administration of PA HCl at 10 mg/kg to the control and 1,25(OH)_2_D_3_-treated rats, the plasma concentration-time profiles of PA and its metabolite NAPA were determined, as shown in Figure 3. The relevant pharmacokinetic parameters determined in the control group (Table 1) were not significantly different from those obtained in a previous study [22]. This suggests that treating rats with the vehicle (i.e., 0.0452% ethanol in filtered corn oil; 1 mL/kg) had no significant effect on the systemic pharmacokinetics of PA and NAPA. Following treatment with 1,25(OH)_2_D_3_ for four consecutive days, however, significant changes in the pharmacokinetic parameters for PA were observed in this study. Regarding the systemic pharmacokinetics, significant decreases in CL and the steady state volume of distribution ($V_{SS}$), along with increased $AUC_{inf}$ were observed in the 1,25(OH)_2_D_3_ treatment group (Table 1). In addition, the $CL_{NR}$ of PA was significantly reduced by 23.4%. However, the $T_{1/2\beta }$ (terminal phase half-life) and mean residence time (MRT) were unchanged by 1,25(OH)_2_D_3_ treatment.

Treatment with 1,25(OH)_2_D_3_ also increased the $AUC_{inf}$ and peak plasma concentration ($C_{max}$) and decreased the $CL_{R}$ of NAPA following the intravenous administration of 10 mg/kg PA HCl. In this study, no significant change was observed in the $T_{1/2\beta }$ and the $AUC_{inf}$ ratio of NAPA to PA ($AUC_{NAPA}/AUC_{PA}$).

### 3.3. Effects of 1,25(OH)_2_D_3_ on the Urinary and Fecal Excretion of PA and NAPA in Rats

**Figure 4:**
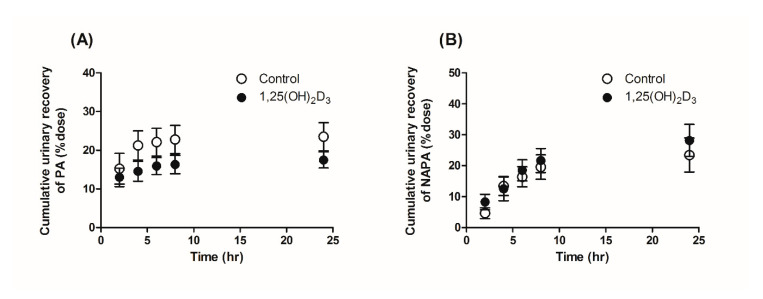
Mean cumulative urinary recovery of (**A**) PA and (**B**) NAPA following intravenous administration of 10 mg/kg PA HCl to control (open circles, *n* = 9) and 1,25(OH)_2_D_3_-treated rats (closed circles, *n* = 7).

To determine the effects of 1,25(OH)_2_D_3_ on the urinary excretion of PA and NAPA, the cumulative urinary recovery of both drugs (% dose) was calculated 24 h after intravenous administration of 10 mg/kg PA HCl. As shown in Figure 4A, the cumulative urinary recovery of PA was significantly lower for the 1,25(OH)_2_D_3_-treated group (i.e., 14.8 and 20.1% for the treated and control groups, respectively), leading to a decrease in $CL_{R}$ (Table 1). In contrast, no significant difference in the cumulative urinary recovery of NAPA was found between the two groups (Figure 4B), while $CL_{R}$ of NAPA decreased by 29.2% following the intravenous administration of PA HCl to the 1,25(OH)_2_D_3_-treated rates due to increased $AUC_{inf}$ following 1,25(OH)_2_D_3_ treatment. PA was not detected in fecal samples, while fecal recovery of NAPA decreased significantly by 47% in the 1,25(OH)_2_D_3_-treated rats compared with the control rats (data not shown). Although NAPA was detected in rat feces, clearance via the fecal pathway was minimal compared to the $CL_{R}$ (i.e., fecal recovery of 1.64% in the control); thus, this was not considered for the current PBPK modeling.

### 3.4. Effects of 1,25(OH)_2_D_3_ on the Tissue Distribution of PA and NAPA at Steady State in Rats

The effects of 1,25(OH)_2_D_3_ on the tissue distribution of PA and NAPA were determined at steady state. $K_{p,ss}$ values of both drugs for the brain, heart, kidneys, liver, lung, and spleen are summarized in Table 2. No statistically significant changes in $K_{p,ss}$ values were observed for the six major tissues following treatment with 1,25(OH)_2_D_3_ (i.e., *p* > 0.05). In general, the observed $K_{p,ss}$ values for the control group (i.e., 1,25(OH)_2_D_3_-free group) fell within a factor of two compared with those reported previously [22], except for the following tissues: liver and lung for PA (i.e., decrease to 15.9% and 36.0%, respectively), and brain, heart, and liver for NAPA (i.e., decrease to 17.8%, 21.0%, and 47.2%, respectively). Due to these discrepancies in the systemic pharmacokinetics of PA and NAPA between the two studies, altered $K_{p,ss}$ values were used for PBPK modeling in the present study.

### 3.5. Effects of 1,25(OH)_2_D_3_ on the In Vitro Metabolic Conversion of PA into NAPA in Rat Liver S9 Fractions

**Figure 5:**
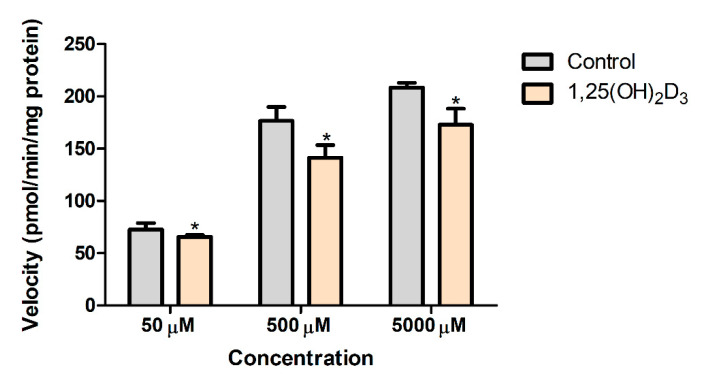
Mean amount of NAPA (pmol/min/mg protein) formed in vitro in S9 fractions of controls and 1,25(OH)_2_D_3_-treated rats (*n* = 4–5). * Indicates *p* < 0.05.

In this study, the amount of NAPA formed in rat liver S9 fractions obtained from the control or the 1,25(OH)_2_D_3_-treated rats was measured. The conversion rate for the treatment group was significantly decreased by 9.5%, 19.9%, and 17.1% at PA concentrations of 50, 500, and 5000 µM, respectively (Figure 5). While the non-renal clearance was reduced by 22.5% in the presence of 1,25(OH)_2_D_3_ treatment (Table 1), a well-stirred assumption of the liver compartment led to decreased $CL_{u,int}$ with a factor of 1.92 (see Section 3.7). As a result, the fraction of NAPA formation during the hepatic elimination of PA ($F_{NAPA}$) increased from 0.562 to 0.845 by 1,25(OH)_2_D_3_ treatment. In addition to the weak change in NAPA formation in the S9 fractions (Figure 5), it was suggested that miscellaneous hepatic elimination of PA may be more significantly affected rather than PA metabolism to NAPA by rNAT-II. Considering that the expression level of rNAT-II enzyme was also markedly reduced by 75.2% based on the qPCR data (Figure 1G), the alteration of mRNA expression level did not appear to quantitatively correlate with the functional change in the metabolic activity for NAPA formation by 1,25(OH)_2_D_3_ treatment.

### 3.6. Free Fraction of PA in the Plasma and the Incubation Mixture of Rat Liver S9 Fractions

The extent of plasma protein binding by PA HCl (5 µg/mL) was compared between the control and 1,25(OH)_2_D_3_-treatment groups. The non-specific binding of PA was 7.52%, suggesting that non-specific binding of the drug to the ultrafiltration membrane or apparatus may be negligible. The free fraction of PA HCl in the plasma of control rats (87.1 ± 0.85%) did not differ from that in the plasma of 1,25(OH)_2_D_3_-treated rats (87.4 ± 3.42%), which is consistent with the results of the previous study [22]. The recovery was 99.4 ± 4.36% and 108 ± 4.11% for the plasma from the control and 1,25(OH)_2_D_3_-treated rats, respectively.

The extent of PA HCl (50 µM) binding to liver S9 fraction protein was compared between the control and 1,25(OH)_2_D_3_-treated rats. The free fraction of PA HCl in the reaction mixture of liver S9 fractions obtained from 1,25(OH)_2_D_3_-treated rats (38.0 ± 0.920%) was not significantly different from that in the reaction mixture of liver S9 fractions obtained from control rats (38.3 ± 3.57%). The recovery was 84.7 ± 5.80% and 88.9 ± 2.88% for the liver S9 fraction from the control and 1,25(OH)_2_D_3_-treated rats, respectively.

### 3.7. Application of a PBPK Model for the Pharmacokinetics of PA and NAPA after Intravenous Administration of PA HCl in the Absence or Presence of 1,25(OH)_2_D_3_ Treatment

**Figure 6:**
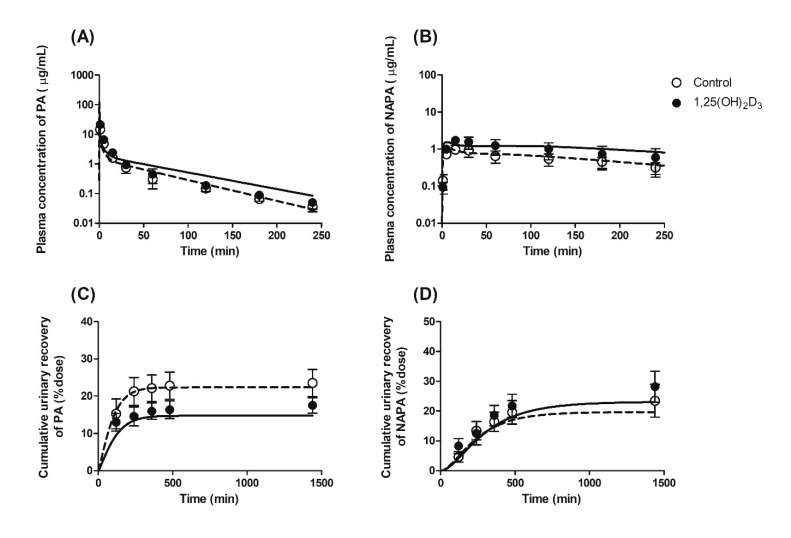
Observed and simulated plasma concentration-time profiles (**A**,**B**) and cumulative urinary excretion profiles (% of dose) (**C**,**D**) for PA (**A**,**C**) and NAPA (**B**,**D**) after intravenous administration of 10 mg/kg PA HCl in the absence or presence of 1,25(OH)_2_D_3_ treatment. Open circles and dashed lines represent the observed and simulated results for the control group, whereas closed circles and solid lines denote the observed and simulated results with the treatment group, respectively. Symbols represent the mean ± S.D.

In this study, a PBPK model [22] was used to elucidate the pharmacokinetics and urinary excretion of PA and NAPA in the absence or presence of 1,25(OH)_2_D_3_. Using the parameters from a series of retrograde calculations (Table 3), the PBPK simulations reasonably captured the observed profiles of the plasma concentration and cumulative urinary excretion in the control group, as shown in Figure 6. When the model parameters in this study are compared with those in the previous report [22], the $K_{p,ss}$ values for kidney (8.484 for PA and 11.86 for NAPA in this study) were observed to be changed from those of the previous literature (5.68 for PA and 21.0 for NAPA). Considering the fraction escaping from the elimination in the kidney (1−ER) consistent between the two studies (i.e., 0.747 for PA and 0.805 for NAPA in this study, and 0.684 for PA and 0.673 for NAPA in the previous study [22]), the observed difference in $K_{p,ss}$ may have resulted from the altered tissue partitioning by the vehicle treatment for four consecutive days that is independent of elimination kinetics. Based on the assumption of a lack of difference in $PS_{out}$, $f_{up}$, and $f_{u,kidney}$ for PA and NAPA in the current kidney model, $PS_{in}$ (22.1 (PA) and 9.59 mL/min (NAPA), Table 3) was also differently estimated from the previous literature (16.2 (PA) and 20.3 mL/min (NAPA)).

In the presence of 1,25(OH)_2_D_3_ treatment, the expression of rOCT1 transporter protein in liver decreased to 30.9% (Figure 2C), consistent with the reduced $K_{p,ss}$ in liver for PA and NAPA (Table 2). However, this decrease was not considered in the current analysis because the in silico prediction of $K_{p}$ based on the tissue binding properties of PA and NAPA [43] overestimated the observed $K_{p}$ values. The protein expression of rMATE1 was reduced to 31.2% of that of the control (Figure 2D), which was used to correct $CL_{u,int,r}$ in the model. Due to indistinguishable $PS_{rOCT1}$ and $PS_{rOCT2}$, the fold change in the overall active uptake ($R_{act}=PS_{act,vitD}/PS_{act}$, Equation (A14)) was determined by fitting our PBPK model to the profiles of plasma concentration and cumulative urinary excretion of PA and NAPA. The fitted pharmacokinetic profiles also adequately described the plasma concentration and urinary excretion profiles of PA and NAPA in the presence of 1,25(OH)_2_D_3_ treatment (Figure 6C,D). Although divergent changes in rOCT1 and rOCT2 expression in kidney (Figure 2D) complicated the kinetic interpretation along with the marked difference in the affinity (e.g., IC50) of PA to both transporters [39,40,41], the estimated $R_{act}$ value of 0.675 (CV% of 61.6) suggested that 1,25(OH)_2_D_3_ treatment in rats may lead to a reduction in the basolateral uptake of PA and NAPA. Based on this calculation, a slight increase (with no statistical significance) in the $K_{p,ss}$ values for PA and NAPA in the kidneys in the presence of 1,25(OH)_2_D_3_ treatment (Table 2) may be ascribed to the reduced apical efflux of PA and NAPA by the rMATE1 transporter. Based on AAFE values within a factor of two for all the plasma and urinary excretion profiles (i.e., 1.43, 1.21, 1.03, and 1.15 from Figure 6A to Figure 6D for the control, and 1.83, 1.58, 1.16, and 1.35 for the treatment group), our PBPK calculation may be generally applicable for the quantitative interpretation of PA and NAPA pharmacokinetics in the presence of 1,25(OH)_2_D_3_ treatment.

## 4. Discussion

VDR, which adopts 1,25(OH)_2_D_3_ as its ligand, regulates the expression of various proteins that may potentially impact the pharmacokinetics of drugs. In human-derived intestinal cell lines (i.e., Caco-2 and LS180), for example, 1,25(OH)_2_D_3_ treatment has been shown to upregulate the mRNA expression of CYP3A4 [44] and increase the expression of P-gp via the VDR pathway [45], which increased the efflux of digoxin from kidney and brain tissues of mice. Increased expression and function of rat multidrug resistance protein 4 (MRP4) following treatment with 1,25(OH)_2_D_3_ [26,46,47] resulted in an increase in the $C_{max}$ (i.e., maximal plasma concentration) and systemic exposure (e.g., AUC) of adefovir (a substrate of rMRP4) via increased basolateral efflux into the blood in rat intestines [25]. In addition, VDR is abundantly expressed in the kidneys, and 1,25(OH)_2_D_3_ treatment was found to decrease renal mRNA levels of rPEPT1, rOAT1, and rOAT3, resulting in a significant decrease in the renal clearance of cefdinir and cefadroxil [12,13]. These findings indicate that changes in the expression of various transporters following treatment with 1,25(OH)_2_D_3_ via VDR activation can lead to changes in the pharmacokinetics of drugs.

In addition to these transporters, OCT and MATE transporters are crucial determinants of the renal elimination kinetics of various drugs [34,35]. Studies have shown that hormonal control is a potential mechanism regulating the expression of OCT transporter(s); for example, rOCT2 expression (both mRNA and protein levels) in the kidneys was increased by testosterone treatment and reduced by estradiol (cf. not for rOCT1 expression), suggesting sex differences in the regulation mechanism of the transporter [48]. Thereafter, the regulation of rOCT2 expression was attributed to involvement of the androgen receptor, which interacts with androgen response element (ARE)-1 and ARE-3 in the rOCT2 promoter region [49]. In addition, steroids, including dexamethasone (2.0-fold), hydrocortisone (2.4-fold), and testosterone (1.8-fold), were found to increase the mRNA expression of endogenous OCT2 in Madin-Darby canine kidney (MDCK) cells [50]. To the best of our knowledge, the present study is the first to report the involvement of another nuclear receptor protein (i.e., VDR) in regulating the mRNA expression of several rOCTs in rat tissues and rMATE1 in the rat kidney.

In this study, a real-time qPCR analysis revealed a significant decrease in renal rOCT2 and rMATE1 mRNA expressions in 1,25(OH)_2_D_3_ treated rats (Figure 1B,D), which were consistent with the altered protein expression (Figure 2D). PA, a substrate of OCT and MATE transporters [34,35], was used as a model drug to investigate the effect of variable expression of the transporters on the renal elimination kinetics of the drug. The results of a previous report showing that active transport accounted for approximately 80% of the total apical uptake of PA by LLC-PK1 cells [51] were consistent with our PBPK model parameters (i.e., $PS_{act}/PS_{in}$ of 65.6%). Consistent with the evidence that the basal mRNA level of renal rOCT2 was 10.3-fold higher than that of renal rOCT1 [52], a lower expression of rOCT1 relative to that of rOCT2 in rat kidneys (38.3 compared to 254 pmol/g kidney) was reported using quantitative proteomics [38]. Considering the higher affinity of PA for rOCT1 than for rOCT2 [39,40,41], the elevated expression level of renal rOCT1 by 1,25(OH)_2_D_3_ treatment (Figure 1) may offset the decreased uptake of PA into rat kidneys by rOCT2. While the protein expression of rOCT2 in kidney was decreased to 34.8% in the control following 1,25(OH)_2_D_3_ treatment (Figure 2D), our model-fitted $R_{act}$ indicated that the overall active uptake of PA and NAPA was reduced to 67.5%, supporting the offsetting effect of the rOCT1 transporter. However, no statistical difference in $K_{p,ss}$ values was observed for PA and NAPA in kidneys between the control and the 1,25(OH)_2_D_3_-treated groups (Table 2). Despite the decrease in overall active uptake of PA into the kidneys, the reduction in renal intrinsic clearance ($CL_{u,int,r}$) due to decreased rMATE1 expression may compensate for the potential reduction in the apparent extent of drug distribution to the kidneys: Our PBPK calculation indicated that the $K_{p,KI}$ values determined from $K_{p,ss}/\left(1-CL_{sec}/Q_{KI}\right)$ (i.e., based on NCA, model-independent) for PA (9.63) and NAPA (15.8) were consistent with the model-based $K_{p,KI}$ values (i.e., calculated by Equation (A12) using the fitted $R_{act}$ value) for PA (10.7) and NAPA (14.5) (Table 3). This suggests that the PBPK model, including the active transport by rOCTs and rMATE1, is useful for understanding the renal disposition kinetics of PA and NAPA. Nevertheless, it may warrant further studies for determining the flipping rate ($k_{cat}$) of rOCT1 and rOCT2 activities for PA and NAPA, which could lead to quantitatively evaluating the contribution of each transporter in the active uptake of these drugs into the kidney.

In the case of the liver, the tissue distribution of PA and NAPA was slightly decreased in the 1,25(OH)_2_D_3_ treatment group, despite no statistical difference in the values (Table 2). In addition, the non-renal clearance ($CL_{NR}$; assumed to be equivalent to hepatic clearance ($CL_{hep}$)) of PA was significantly decreased in the presence of 1,25(OH)_2_D_3_ treatment. We reasoned that these phenomena may be explained in part by reduced expression of rOCT1 and rNAT-II in the liver (e.g., in terms of mRNA, 0.68- and 0.248-fold expression versus the control, Figure 1). According to the extended clearance concept [53], the uptake clearance ($PS_{in}$) is proportional to the apparent intrinsic clearance (i.e., $CL_{u,int,all}=PS_{in}\times CL_{u,met}/\left(PS_{out}+CL_{u,met}\right)=PS_{in}\times \beta$), and thus, the decrease of $PS_{in}$ and $CL_{u,met}$ may also result in the reduction of $CL_{u,int,all}$. Assuming that a portion of the saturable component in the hepatic uptake of PA-ethobromide is equivalent to that of PA (i.e., 55.7%) [54] and the observed 0.309-fold change in the protein level of hepatic rOCT1 (Figure 2C) is directly applicable to functional change in the transporter, the calculated hepatic $PS_{in}$ was decreased by 38.5%. However, the in silico $K_{p}$ predictions based on partitioning properties to tissue constituents [43] resulted in a value of 8.02 for both PA and NAPA, suggesting that additional mechanisms may be involved in the liver distribution of these drugs. Therefore, although reduced expression of hepatic rOCT1 and rNAT-II may provide insight into the significant reduction in $CL_{NR}$ of PA and NAPA, further studies are needed to understand the potential involvement of the basolateral efflux of the drugs back into the blood circulation. In addition, although the level of rOCT3 mRNA expression in the heart decreased following 1,25(OH)_2_D_3_ treatment (Figure 1), the tissue distribution of PA and NAPA to heart tissue appeared unchanged in this study.

Based on the in vitro metabolism study in liver S9 fractions, the use of 50 µM concentration in the control resulted in 1.45 µL/min/mg protein. Considering S9 protein per gram liver of 135 mg/g liver and 9 g liver (obtained from Simcyp V19 Release 1; Simcyp Ltd., Certara Co., Sheffield, UK) [55], the unbound intrinsic formation clearance corrected by the free fraction of PA in the incubation mixture (i.e., $f_{u,inc}=$ 0.380) was calculated to be 4.64 mL/min. In terms of the in vitro–in vivo extrapolation (IVIVE) of PBPK parameters, an additional scaling factor of 5.80, was needed to describe the unbound intrinsic formation clearance ($CL_{u,int}\cdot F_{NAPA}$ of 26.9 mL/min, Table 3). Moreover, the increase in $F_{NAPA}$ following 1,25(OH)_2_D_3_ treatment, as well as the weak reduction of NAPA formation in the S9 fractions (Figure 5), suggested that the miscellaneous hepatic elimination of PA may be more significantly affected rather than PA metabolism to NAPA by rNAT-II. Despite the practical utility of our PBPK model, further studies are warranted to examine the unaccounted factors affecting the altered pharmacokinetics of PA and NAPA.

In this study, we aimed to elucidate the effect of 1,25(OH)_2_D_3_ on the expression of rOCTs and rMATE1 transporters and hence the pharmacokinetics of their substrate drugs, PA and NAPA. Since the current PBPK model was useful for linking the transporter activity with renal elimination kinetics for the drugs in rat, this consideration may be also applicable for assessing the potential alteration in the pharmacokinetics of the substrate drugs for OCTs and MATE1 in man, and thus for predicting unexpected toxicity and maximizing drug efficacy when taking the related medicines in the presence of 1,25(OH)_2_D_3_. Based on a lot of clinical observations showing that renal transporters play an important role on drug elimination and systemic exposures, further research on the alterations in OCTs and MATE1 expressions depending on the 1,25(OH)_2_D_3_ level in different clinical settings would be interesting.

Although this is the first study to report the possible involvement of VDR in the regulation of organic cation transporters, the mechanism underlying this regulation requires further investigation to elucidate the different effects of VDR among the tissues (i.e., OCT1 increased in kidney and decreased in liver) or the transporters (i.e., increased rOCT1 and decreased rOCT2 in kidney). The determination of VDR binding sites for the gene of each transporter or the investigation of the profiles/property of VDR translocation may provide important insight into the detailed mechanisms underlying organic cation transporter regulation.

## 5. Conclusions

To our knowledge, the current study is the first to reveal that 1,25(OH)_2_D_3_ treatment affects the expression levels of OCT isoforms and MATE transporters in rats, suggesting VDR as a regulating mechanism for the proteins. The mRNA/protein expression of rOCT1 was significantly increased in the kidneys of 1,25(OH)_2_D_3_-treated rats compared with control rats, whereas the mRNA or protein levels of rOCT2 and rMATE1 in the kidney, rOCT1 and rNAT-II in the liver, and rOCT3 in the heart were significantly decreased. In addition, 1,25(OH)_2_D_3_ treatment resulted in a significant decrease in the systemic CL of PA, a substrate of rOCT2 and rMATE1. The diminished renal clearance of PA and NAPA was successfully addressed by decreased rOCT2 and rMATE1 expression levels in the kidney following 1,25(OH)_2_D_3_ treatment, using a PBPK model for PA and NAPA. A physiological model for the pharmacokinetics of PA and NAPA in rats was useful for linking changes in the transcription and expression of the rOCTs and rMATE1 transporters to the altered pharmacokinetic of the drugs.

## Figures and Tables

**Table 1:** Pharmacokinetic parameters of PA and NAPA following intravenous administration of 10 mg/kg PA HCl to control and 1,25(OH)_2_D_3_-treated rats.

Parameter	Control (*n* = 9)	1,25(OH)_2_D_3_ (*n* = 9)
Initial body weight (g)	309.1 ± 15.3	326.1 ± 14.7
Final body weight (g)	297.6 ± 19.3	282.3 ± 16.0 **
**Procainamide**		
$AUC_{inf}$ (µg·min/mL)	146.6 ± 19.4	208.5 ± 42.1 *
$T_{1/2\beta }$ (min)	52.01 ± 5.9	68.09 ± 22.95
MRT (min)	31.62 ± 5.50	33.05 ± 4.46
$V_{SS}$ (mL/kg)	2153 ± 432	1616 ± 236 *
CL (mL/min/kg)	69.24 ± 8.95	49.72 ± 9.88 **
$CL_{R}$ (mL/min/kg)	16.29 ± 2.11	8.696 ± 1.728 **
$CL_{NR}$ (mL/min/kg)	52.95 ± 6.85	41.02 ± 8.15 *
**N-acetylprocainamide**		
$AUC_{inf}$ (µg·min/mL)	196.7 ± 57.9	402.5 ± 228.6 *
$T_{1/2\beta }$ (min)	132.3 ± 24.6	151.6 ± 49.4
$C_{max}$ (µg/mL)	0.9958 ± 0.2004	1.740 ± 0.476 **
$T_{max}$ (min)	23.33 ± 7.91	16.67 ± 5.00 *
$CL_{R}$ (mL/min/kg)	12.67 ± 2.96	8.971 ± 4.241 *
$AUC_{NAPA}/AUC_{PA}$	1.328 ± 0.256	1.853 ± 0.799

Key: $AUC_{inf}$, area under the plasma concentration-time curve from time zero to infinity; MRT, mean residence time; $T_{1/2\beta }$, terminal phase half-life; $V_{SS}$, apparent volume of distribution at steady state; CL, total clearance; $CL_{R}$, renal clearance; $CL_{NR}$, non-renal clearance; $C_{max}$, peak plasma concentration; $T_{max}$, time to reach $C_{max}$. * *p* < 0.05 and ** *p* < 0.001 between control and 1,25(OH)_2_D_3_-treated rats.

**Table 2:** Tissue-to-plasma partition coefficient at steady state ($K_{p,ss}$) for PA and NAPA in major tissues from control and 1,25(OH)_2_D_3_-treated rats. Data are represented as the mean ± S.D. (*n* = 5).

Tissue	PA			NAPA		
	Control	1,25(OH)_2_D_3_	*p* Value	Control	1,25(OH)_2_D_3_	*p* Value
Brain	0.3904 ± 0.0917	0.2861 ± 0.0646	0.0714	0.1268 ± 0.0457	0.2458 ± 0.1361	0.1010
Heart	2.362 ± 0.912	1.658 ± 0.217	0.1317	2.670 ± 0.248	2.290 ± 0.689	0.2794
Kidney	8.484 ± 1.671	8.775 ± 1.078	0.7517	11.86 ± 4.30	13.93 ± 3.67	0.4360
Liver	0.4561 ± 0.1680	0.3059 ± 0.0605	0.0968	8.410 ± 4.092	6.338 ± 2.285	0.3517
Lungs	0.9077 ± 0.1073	0.9282 ± 0.0511	0.7092	5.448 ± 1.140	4.191 ± 1.130	0.1180
Spleen	2.027 ± 0.329	1.507 ± 0.454	0.0718	6.432 ± 0.958	5.186 ± 1.825	0.2137

**Table 3:** Input parameters for PBPK modeling of PA and NAPA in the absence or presence of 1,25(OH)_2_D_3_ treatment.

Parameter	Control		1,25(OH)_2_D_3_ Treatment		Comment
	PA	NAPA	PA	NAPA	
**Physchem and Blood Binding**					
Molecular weight	235.33	277.36	235.33	277.36	
Compound type	Monoprotic base		Monoprotic base		
pKa	9.04	9.04	9.04	9.04	
log P	0.83	0.93	0.83	0.93	
$f_{up}$	0.87	0.688	0.87	0.688	
B/P ratio	1	1	1	1	
**Distribution ($K_{p}$)**					
Kidney	11.4	14.7	10.7 ^b^	14.5 ^b^	$K_{p,ss}/\left(1-ER\right)$
Liver	1.44	9.62	0.649	7.25	$K_{p,ss}/\left(1-ER\right)$
Brain	0.3904	0.1268	0.2861	0.2458	$K_{p,ss}$
Heart	2.362	2.67	1.658	2.29	$K_{p,ss}$
Lung	0.9077	5.448	0.9282	4.191	$K_{p,ss}$
Spleen	2.027	6.432	1.507	5.186	$K_{p,ss}$
Adipose	0.721	0.967	0.721	0.967	Predicted [43]
Bone	1.96	2.2	1.96	2.2	Predicted [43]
Gut	4.87	6.23	4.87	6.23	Predicted [43]
Muscle	3.93	4.61	3.93	4.61	Predicted [43]
Skin	2.96	3.64	2.96	3.64	Predicted [43]
**Non-Renal Elimination**					
$CL_{u,int}$ (mL/min) ^a^	47.9	4.04	25	4.04	$\frac{Q_{LI}\;\cdot \;CL_{NR}}{f_{up}\;\cdot \;\left(Q_{LI}-CL_{NR}\right)}$
Extraction ratio ^a^	0.682	0.125	0.529	0.125	$CL_{NR}/Q_{LI}$
$CL_{int,m}$ (mL/min)	16.3		28.3		$CL_{m}/K_{p,ss,LI}$
$F_{NAPA}$ ^a^	0.562		0.845		$CL_{int,m}/CL_{int}$
**Semi-Mechanistic Kidney**					
$CL_{u,int,r}$ (mL/min)	4.67	9.16	1.46 ^c^	2.86 ^c^	From the previous model [22]
$PS_{out}$ (mL/min)	7.61	7.61	7.61	7.61	From the previous model [22] ($=PS_{pas}$)
$PS_{in}$ (mL/min)	22.1	9.59	17.4 ^d^	8.95 ^d^	$PS_{act}+PS_{pas}$
$CL_{rabs}$ (mL/min)	0.415	0.415	0.415	0.415	From the previous model [22]
Extraction ratio ^a^	0.253	0.195	0.0892	0.116	$CL_{sec}/Q_{KI}$
$f_{u,kidney}$	0.223	0.0588	0.223	0.0588	From the previous model [22]

^a^ Determined from non-compartmental analysis of the observed data; ^b^ Model-based $K_{p,KI}$ values were calculated using the relationship $K_{p,KI}=\frac{f_{up}PS_{in}}{f_{u,kidney}PS_{out}}$ (Equation (A12)); ^c^ For 1,25(OH)_2_D_3_ treatment group, $CL_{u,int,r,vitD}=CL_{u,int,r}\cdot R_{MATE}$, where $R_{MATE}=$ 0.312 from protein expression data; ^d^ Using the fitted $R_{act}$ value (0.675, with Coefficient of Variation of 61.6%), the uptake clearance into the proximal tubule cell compartment in the presence of 1,25(OH)_2_D_3_ treatment ($PS_{in,vitD}$) was calculated by $PS_{in,vitD}=PS_{act,vitD}+PS_{pas}=PS_{act}R_{act}+PS_{pas}$.

## Data Availability

The data presented in this study are available in the article.

## Supplementary Materials

The following are available online at https://www.mdpi.com/article/10.3390/pharmaceutics13081133/s1, ADAPT model code used for estimating $R_{act}$ is provided in the Supplementary Material.

## Appendix A

In the previous study [22], a whole-body PBPK model was proposed for the pharmacokinetics of PA and NAPA in rats, and the same model structure was utilized in this study along with slightly modified parameter values. Based on the perfusion-limited model [22,56], the differential equation for non-eliminating organs (i.e., tissues except for kidney and liver) may be expressed as follows (Equation (A1)):

(A1) $$V_{T}\frac{dC_{T}}{dt}=Q_{T}\cdot \left(C_{art}-\frac{C_{T}\cdot R}{K_{p}}\right)$$

where $V_{T}$ is the volume of tissue compartment; $C_{T}$ and $C_{art}$ are the drug concentrations in the tissue and arterial blood compartments, respectively; $Q_{T}$ is the blood flow to the tissue; R is the blood-to-plasma concentration ratio; $K_{p}$ is the equilibrium tissue-to-plasma concentration ratio.

For liver tissue compartment (Equation (A2)):

(A2) $$V_{LI}\frac{dC_{LI}}{dt}=\left(Q_{LI}-Q_{GU}-Q_{SP}\right)\cdot C_{art}+Q_{GU}\frac{C_{GU}\cdot R}{K_{p,GU}}+Q_{SP}\frac{C_{SP}\cdot R}{K_{p,SP}}-Q_{LI}\frac{C_{LI}\cdot R}{K_{p,LI}}-CL_{u,int}\frac{f_{up}}{K_{p,LI}}C_{LI}$$

where $V_{LI}$ is the volume of the liver; $C_{LI}$, $C_{GU}$, and $C_{SP}$ are the drug concentrations in the liver, gut, and spleen, respectively; $Q_{LI}$, $Q_{GU}$, and $Q_{SP}$ are the blood flow to the liver, gut, and spleen, respectively; $K_{p,LI}$, $K_{p,GU}$, and $K_{p,SP}$ are the equilibrium tissue-to-plasma concentration ratios for the liver, gut, and spleen, respectively; and $CL_{u,int}$ is the intrinsic clearance of drug molecules in the liver compartment.

In the venous blood compartment (i.e., dosing compartment), (Equation (A3)):

(A3) $$V_{ven}\frac{dC_{ven}}{dt}=Q_{AD}\frac{C_{AD}\cdot R}{K_{p,AD}}+Q_{BO}\frac{C_{BO}\cdot R}{K_{p,BO}}+Q_{BR}\frac{C_{BR}\cdot R}{K_{p,BR}}+Q_{HE}\frac{C_{HE}\cdot R}{K_{p,HE}}+Q_{LI}\frac{C_{LI}\cdot R}{K_{p,LI}}\;+Q_{MU}\frac{C_{MU}\cdot R}{K_{p,MU}}+Q_{SK}\frac{C_{SK}\cdot R}{K_{p,SK}}+(Q_{KI}-Q_{U})\cdot C_{RBL}+Q_{RE}\cdot C_{art}\;-Q_{CO}\cdot C_{ven}-Dose\;rate$$

where $V_{ven}$ is the volume of venous blood; $C_{AD}$, $C_{BO}$, $C_{BR}$, $C_{HE}$, $C_{MU}$, $C_{SK}$, $C_{RBL}$, and $C_{ven}$ are the drug concentrations in the adipose, bone, brain, heart, muscle, skin, renal blood, and venous blood compartments, respectively; $Q_{AD}$, $Q_{BO}$, $Q_{BR}$, $Q_{HE}$, $Q_{MU}$, $Q_{SK}$, $Q_{KI}$, and $Q_{RE}$ are the blood flows to the adipose, bone, brain, heart, muscle, skin, and kidney and the residual blood flow, respectively; $Q_{U}$ and $Q_{CO}$ are the urinary flow and cardiac output, respectively; and $K_{p,AD}$, $K_{p,BO}$, $K_{p,BR}$, $K_{p,HE}$, $K_{p,MU}$, and $K_{p,SK}$ are the equilibrium tissue-to-plasma concentration ratio of adipose, bone, brain, heart, muscle, and skin, respectively. Dose rate is the dosing rate of drugs to the venous blood.

In the lung compartment, (Equation (A4)):

(A4) $$V_{LU}\frac{dC_{LU}}{dt}=Q_{CO}\cdot \left(C_{ven}-\frac{C_{LU}\cdot R}{K_{p,LU}}\right)$$

where $V_{LU}$ is the volume of the lung; $C_{LU}$ is the drug concentration in the lung; $K_{p,LU}$ is the equilibrium tissue-to-plasma concentration ratio for lung.

In the arterial blood compartment (Equation (A5)):

(A5) $$V_{art}\frac{dC_{art}}{dt}=Q_{CO}\cdot \left(\frac{C_{LU}\cdot R}{K_{p,LU}}-C_{art}\right)$$

where $V_{art}$ is the volume of arterial blood.

In addition, a semi-mechanistic kidney model was incorporated into the PBPK model to predict the effect of the altered expressions of renal transporters on the pharmacokinetics of PA/NAPA in terms of basolateral uptake and apical efflux. We used the same values of physiological input parameters as applied in the previous study [22]. It was noted that the pharmacokinetic variables in the differential equations, especially for semi-mechanistic kidney compartments, are applied for each compound (e.g., $f_{up}$, R, and $K_{p,KI}$). Since drug molecules delivered to rat glomerulus may be drained with a filtration rate of $f_{up}GFR/R$, the differential equation for the glomerulus is expressed as follows (Equation (A6)):

(A6) $$V_{GLM}\frac{dC_{GLM}}{dt}=Q_{KI}\cdot C_{art}-f_{up}GFR/R\cdot C_{GLM}-\left(Q_{KI}-f_{up}GFR/R\right)\cdot C_{GLM}$$

where $C_{GLM}$ is the drug concentration in the glomerulus. Fluid reabsorption from the three S1 segments of proximal tubules was described as follows (Equations (A7)–(A9)):

(A7) $$V_{S1\_1}\frac{dC_{S1\_1}}{dt}=f_{up}GFR/R\cdot C_{GLM}-Q_{S1\_2}\cdot C_{S1\_1}$$

(A8) $$V_{S1\_2}\frac{dC_{S1\_2}}{dt}=Q_{S1\_2}\cdot C_{S1\_1}-Q_{S1\_3}\cdot C_{S1\_2}$$

(A9) $$V_{S1\_3}\frac{dC_{S1\_3}}{dt}=Q_{S1\_3}\cdot C_{S1\_2}-Q_{S2+S3}\cdot C_{S1\_3}$$

where $C_{S1\_1}$, $C_{S1\_2}$, and $C_{S1\_3}$ are the drug concentration in the first, second, and third compartments of the S1 segment of proximal tubules, respectively. The renal secretion and reabsorption processes were assumed to occur in the S2 and S3 segments of proximal tubules as follows (Equation (A10)):

(A10) $$V_{S2+S3}\frac{dC_{S2+S3}}{dt}=Q_{S2+S3}\cdot C_{S1\_3}-Q_{LOH}\cdot C_{S2+S3}+CL_{u,int,r}\cdot f_{u,kidney}\cdot C_{PTC}-CL_{rabs}\cdot C_{S2+S3}$$

where $C_{S2+S3}$ and $C_{PTC}$ are the drug concentrations in the S2 and S3 segments and proximal tubule cell compartment, respectively; $CL_{u,int,r}$ is the renal intrinsic clearance of drugs from the proximal tubule cell compartment into the S2 and S3 segments; $CL_{rabs}$ is the reabsorption clearance of drugs into the proximal tubule cell compartment; and $f_{u,kidney}$ is the free fraction of drugs in kidney cells (i.e., renal proximal tubule cells).

The remaining fraction of drugs avoiding glomerular filtration was considered to be delivered to a renal blood compartment, in which drug molecules are transported into and out of the proximal tubule cell compartment (Equation (A11)):

(A11) $$V_{RBL}\frac{dC_{RBL}}{dt}=\left(Q_{KI}-f_{up}GFR/R\right)\cdot C_{GLM}-\left(Q_{KI}-Q_{U}\right)\cdot C_{RBL}-f_{up}PS_{in}/R\cdot (C_{RBL}-\frac{C_{PTC}\cdot R}{K_{p,KI}})$$

where $C_{PTC}$ is the drug concentration in the proximal tubule cell compartment, and $K_{p,KI}$ is the equilibrium tissue-to-plasma concentration ratio, which could be also expressed as the following (Equation (A12)):

(A12) $$K_{p,KI}=\frac{f_{up}PS_{in}}{f_{u,kidney}PS_{out}}$$

where $PS_{in}$ and $PS_{out}$ are the tissue permeabilities of drugs into and out of proximal tubule cells, respectively. Essentially, $K_{p,uu}$ can be calculated as the ratio of $PS_{in}$ to $PS_{out}$. $PS_{in}$ may be involved with the active ($PS_{act}$) and passive transport ($PS_{pas}$):

(A13) $$K_{p,KI}=\frac{PS_{in}}{PS_{out}}K_{p,KI,pass}=\frac{PS_{act}+PS_{pas}}{PS_{pas}}K_{p,KI,pass}$$

where $K_{p,KI,pass}$ is the tissue partitioning coefficient only by tissue binding (i.e., symmetrical passive transport) and thus the ratio of $f_{up}$ to $f_{u,kidney}$ [43]. The drug efflux from the proximal tubule cells to the blood was assumed to be dependent on the passive diffusion across the basolateral membrane ($PS_{out}$). Due to the indistinguishable $PS_{rOCT1}$ and $PS_{rOCT2}$ (see main text), the effect of 1,25(OH)_2_D_3_ treatment on $PS_{act}$ was described as the overall fold-difference ($R_{act}$):

(A14) $$R_{act}=PS_{act,vitD}/PS_{act}$$

For the proximal tubule cell compartment, the differential equation may be described as follows (Equation (A15)):

(A15) $$V_{PTC}\frac{dC_{PTC}}{dt}=f_{up}PS_{in}/R\cdot (C_{RBL}-\frac{C_{PTC}\cdot R}{K_{p,KI}})-CL_{u,int,r}\cdot f_{u,kidney}\cdot C_{PTC}+CL_{rabs}\cdot C_{S2+S3}$$

After two third of fluid was reabsorbed from the proximal tubules, one-third of the remaining fluid enters the Loop-of-Henle, in which 15% of the filtered fluid is reabsorbed, as described in Equation (A16):

(A16) $$V_{LOH}\frac{dC_{LOH}}{dt}=Q_{LOH}\cdot C_{S2+S3}-Q_{DT+CD}\cdot C_{LOH}$$

where $C_{LOH}$ is the drug concentration in the Loop-of-Henle. As described in previous literature [37,57], the kidney model used in this study assumes that the lumen of the distal nephron segments mainly consists of the distal tubules and collecting ducts, which are considered to be kinetically indistinguishable, and receives approximately 18% of the filtered fluid. Since about 16% of fluid reabsorption of the total filtrate was known to occur from this compartment, the urine flow rate ($Q_{U}$) of 2% of the filtration rate was considered as described below (Equations (A17)–(A19)) [58,59,60]:

(A17) $$V_{DT+CD}\frac{dC_{DT+CD}}{dt}=Q_{DT+CD}\cdot C_{LOH}-Q_{U}\cdot C_{DT+CD}$$

(A18) $$V_{U}\frac{dC_{U}}{dt}=Q_{U}\cdot C_{DT+CD}-Q_{U}\cdot C_{U}$$

(A19) $$\frac{dA_{E}}{dt}=Q_{U}\cdot C_{U}$$

For the case of liver and kidney, $K_{p,ss}$ was corrected to the equilibrium tissue-to-plasma partition coefficient ($K_{p}$), using the following relationship [61] (Equation (A20)):

(A20) $$K_{p}=\frac{K_{p,ss}}{1-ER}$$

where ER is the extraction ratio, which could be calculated as the ratio of hepatic (i.e., equivalent to non-renal clearance, $CL_{H}=CL_{NR}=CL-CL_{R}$) or renal secretion clearance (i.e., $CL_{SEC}=CL_{R}-f_{up}GFR$) to blood perfusion rate to the liver or kidney ($Q_{LI}$ or $Q_{KI}$).

The AUC values for the plasma concentration of PA ($AUC_{PA}$) and NAPA ($AUC_{NAPA}$) after the intravenous administration of PA were used to calculate the apparent formation clearance ($CL_{m}$) from PA to NAPA using the following relationship (Equation (A21)):

(A21) $$CL_{m}=\frac{AUC_{NAPA}}{AUC_{PA}}CL_{\left(m\right)}$$

where $CL_{\left(m\right)}$ is the disposition clearance of NAPA (22.4 mL/min/kg for the control group) [62]. Due to the absence of direct measurement of non-renal clearance of NAPA, $CL_{\left(m\right)}$ for the 1,25(OH)_2_D_3_ treatment group was considered to be 18.7 mL/min/kg, based on the assumption that the change in $CL_{\left(m\right)}$ is only dependent on the alteration of renal clearance of NAPA. Assuming that PA is metabolized to NAPA only in liver, the intrinsic formation clearance from PA to NAPA in the liver ($CL_{int,m}$, with respect to the liver concentration of PA) was calculated as follows (Equation (A22)):

(A22) $$CL_{int,m}=\frac{AUC_{PA}}{AUC_{PA,LI}}CL_{m}=\frac{CL_{m}}{K_{p,ss,LI}}$$

where $AUC_{PA,LI}$ is the area under the liver concentration curve of PA; $K_{p,ss,LI}$ is the steady-state liver-to-plasma concentration ratio. Total hepatic intrinsic clearance of PA ($CL_{int}$) was calculated based on the well-stirred assumption of liver compartment (Equation (A23)):

(A23) $$CL_{int}=\frac{Q_{LI}\cdot CL_{H}}{\left(Q_{LI}-CL_{H}\right)/K_{p,LI}}$$

where $K_{p,LI}$ is the equilibrium tissue-to-plasma partition coefficient corrected from $K_{p,ss,LI}$. The fraction of NAPA formation ($F_{NAPA}$) during the hepatic elimination of PA was then calculated as the ratio of $CL_{int,m}$ to $CL_{int}$. All the parameters necessary for PBPK calculations in accordance with the previous model are summarized in Table 3.
