# Supplementary material for: Effects of 1α,25-Dihydroxyvitamin D3 on the Pharmacokinetics of Procainamide and Its Metabolite N-Acetylprocainamide, Organic Cation Transporter Substrates, in Rats with PBPK Modeling Approach
Source: Pharmaceutics. 2021 Jul 25;13(8):1133. doi: 10.3390/pharmaceutics13081133 (PMC8402143; doi:10.3390/pharmaceutics13081133)
Supplement: Supplementary file 1 [file pharmaceutics-13-01133-s001.zip › pharmaceutics-1276423-supplementary.pdf]

pharmaceutics

Supplementary Material: Effects of 1α,25-Dihydroxyvitamin D_3 on the Pharmacokinetics of Procainamide and Its Metabolite N-Acetylprocainamide, Organic Cation Transporter Substrates, in Rats with PBPK Modeling Approach

Anusha Balla, Yoo-Seong Jeong, Hyojung Kim, Yunjong Lee, Suk-Jae Chung, Yoon-Jee Chae and Han-Joo Maeng

```
*********************************************************************
C                          ADAPT                              *
C                        Version 5                            *
C*********************************************************************
C                                                             *
C                          MODEL                              *
C                                                             *
C    This file contains Fortran subroutines into which the user       *
C    must enter the relevant model equations and constants.           *
C    Consult the User's Guide for details concerning the format for   *
C    entered equations and definition of symbols.                     *
C                                                             *
C       1. Symbol-   Parameter symbols and model constants            *
C       2. DiffEq-   System differential equations                    *
C       3. Output-   System output equations                          *
C       4. Varmod-   Error variance model equations                   *
C       5. Covmod-   Covariate model equations (ITS,MLEM)             *
C       6. Popinit- Population parameter initial values (ITS,MLEM)    *
C       7. Prior -   Parameter mean and covariance values (ID,NPD,STS) *
C       8. Sparam-   Secondary parameters                             *
C       9. Amat   -   System state matrix                             *
C                                                             *
C*********************************************************************
C######################################################################C
        Subroutine SYMBOL
        Implicit None
        Include 'globals.inc'
        Include 'model.inc'
CC
C----------------------------------------------------------------------C
C    Enter as Indicated                                               C
C----c-----------------------------------------------------------------C
      NDEqs    =   46    ! Enter # of Diff. Eqs.
```

```
        NSParam =   1     ! Enter # of System Parameters.
        NVparam =   2     ! Enter # of Variance Parameters.
        NSecPar =   0     ! Enter # of Secondary Parameters.
        NSecOut =   0   ! Enter # of Secondary Outputs (not used).
        Ieqsol   =   1   ! Model type: 1 - DIFFEQ, 2 - AMAT, 3 - OUTPUT only.
        Descr     = ' Insert Model File Description    '
CC
C----------------------------------------------------------------------C
C     Enter Symbol for Each System Parameter (eg. Psym(1)='Kel')                C
C----c-----------------------------------------------------------------C
        Psym(1) = 'ROCT'
CC
C----------------------------------------------------------------------C
C     Enter Symbol for Each Variance Parameter {eg: PVsym(1)='Sigma'}       C
C----c-----------------------------------------------------------------C
        PVsym(1)='Sigma'
        PVsym(2)='Intercept'
CC
C----------------------------------------------------------------------C
C     Enter Symbol for Each Secondary Parameter {eg: PSsym(1)='CLt'}         C
C----c-----------------------------------------------------------------C
C----------------------------------------------------------------------C
C----------------------------------------------------------------------C
C
          Return
          End
C######################################################################C
          Subroutine DIFFEQ(T,X,XP)
          Implicit None
          Include 'globals.inc'
          Include 'model.inc'
          Real*8 T,X(MaxNDE),XP(MaxNDE)
          Real*8 RMATE, CLintK, NCLintK, FORM, CLintL, NCLintL
          Real*8 QG, Q, QL, QB, QK, QH, QS, QI, QM, QA, QSk, QBo, QC
          Real*8 VL, VB, VK, VH, VG, VS, VI, VM, VA, VSk, VBo, VVE, VAR
          Real*8 SK, PP, PK, NPK
          Real*8 KpL, KpG, KpB, KpH, KpK, KpM, KpS, KpI, KpA, KpBo, KpSk
          Real*8 NKpL, NKpG, NKpB, NKpH, NKpK, NKpM, NKpS, NKpI, NKpA
          Real*8 Nfup, fup, GFR, NKpBo, NKpSk
          Real*8 QS2, QS3, QS4, Qloh, QCD, QU, FractionGFRVol
          Real*8 Vglm, Vrbl, VS1, VS2, VS3, VS4, Vloh, Vcd, Vu, Vptc
          Real*8 CLrabs, NCLrabs, MW, NMW, Dose, ROCT
```

```
CC
C---------------------------------------------------------------------C
C    Enter Differential Equations Below    {e.g.    XP(1) = -P(1)*X(1) }        C
C----c-----------------------------------------------------------------C
           ROCT = P(1)

           RMATE = 0.312
           CLintK = 4.67 * RMATE
           NCLintK = 9.16 * RMATE
           FORM = 0.845
           QG=80
           Q=80
           QL=80*24.2/100
           QB=80*1.4/100
           QK=80*14.5/100
           QH=80*4/100
           QS=80*1.1/100
           QI=80*10.1/100
           QM=80*23.7/100
           QA=80*5.9/100
           QSk=80*5.1/100
           QBo=80*10.1/100

           VL=8.57
           VB=1.24
           VK=2.19
           VH=1.05
           VG=1.24
           VS=0.57
           VI=6.19
           VM=116.13
           VA=16.66
           VSk=39.4
           VBo=15.7
           VVE=15.32*2/3
           VAR=15.32*1/3

           SK=397000
           PP=0.00000031
           PK= (0.0000009 - PP)*ROCT + PP
           NPK = (0.000000391 - PP)*ROCT + PP
           KpL = 0.649
```

KpG = 0.9282
KpB = 0.2861
KpH = 1.6583
KpK = PK/PP*3.91
KpM = 3.93
KpS = 1.507
KpI = 4.87
KpA = 0.721
KpBo = 1.96
KpSk = 2.96

NKpL=7.25
NKpG = 4.191
NKpB =0.2458
NKpH = 2.290
NKpK = NPK/PP*11.7
NKpS =5.186
NKpI = 6.23
NKpA = 0.967
NKpBo = 2.2
NKpSk = 3.64
NKpM =4.61
Nfup = 0.688
fup=0.87
GFR = 1.31

CLintL=25
NCLintL=4.04

QC=Q-(QK+QL+QB+QH+QM+QA+QSK+QBO)

QS2 = GFR*0.85
QS3 = GFR*0.70
QS4 = GFR*0.55
Qloh = GFR*0.33
QCD = GFR*0.18
QU = GFR*0.02
FractionGFRVol = GFR/QK
Vglm = 0.08
Vrbl = 0.375
VS1 = Vglm*FractionGFRVol
VS2 = Vglm*FractionGFRVol*0.85

```
      VS3 = Vglm*FractionGFRVol*0.70
      VS4 = Vglm*FractionGFRVol*0.55
      Vloh = Vglm*FractionGFRVol*0.33
      Vcd =   Vglm*FractionGFRVol*0.18
      Vu = 1.44
      Vptc = 1.03
      CLrabs = 0.96*Qloh
      NCLrabs = 0.96*Qloh
      MW=235.325
      NMW=277.362

      XP(1) = (   QC*X(3) + QL*X(4)/KpL + QB*X(5)/KpB + (QK-QU)*X(19)
     1 +QH*X(7)/KpH + QM*X(10)/KpM + QA*X(11)/KpA + QSk*X(12)/KpSk
     1 + QBo*X(13)/KpBo -QG*X(1) )/VVE
      XP(2) = QG*(X(1)-X(2)/KPG)/VG
      XP(3) = QG*(X(2)/KPG-X(3))/VAR
      XP(4) = (QL*(X(3)-( QS*(X(3)-X(8)/KPS)+ QI*(X(3)-X(9)/KPI))/(QL)
     1 -X(4)/KpL)-CLintL*fup/KpL*X(4))/VL
      XP(5) = QB*(X(3)-X(5)/KPB)/VB
      XP(6) = (fup*PK*60*SK*Vptc* ( X(19) - X(6)/KpK ) + CLrabs*X(18)
     1 - CLintK*fup/3.91*X(6))/Vptc
      XP(7) = QH*(X(3)-X(7)/KPH)/VH
      XP(8) = QS*(X(3)-X(8)/KPS)/VS
      XP(9) = QI*(X(3)-X(9)/KPI)/VI
      XP(10) = QM*(X(3)-X(10)/KPM)/VM
      XP(11) = QA*(X(3)-X(11)/KPA)/VA
      XP(12) = QSk*(X(3)-X(12)/KPSk)/VSk
      XP(13) = QBo*(X(3)-X(13)/KPBo)/VBo
      XP(14) = QK*(X(3)-X(14))/Vglm
      XP(15) = ( fup*GFR*X(14) - QS2*X(15) ) / VS1
      XP(16) = ( QS2*X(15) - QS3*X(16) ) / VS2
      XP(17) = ( QS3*X(16) - QS4*X(17) ) / VS3
      XP(18) = ( QS4*X(17) - Qloh*X(18) + CLintK*fup/3.91*X(6)
     1 - CLrabs*X(18) ) / VS4
      XP(19) = (( QK - fup*GFR )*X(14) - fup*PK*60*SK*Vptc* ( X(19)
     1 - X(6)/KpK ) - (QK-QU) * X(19) ) / Vrbl
      XP(20) = ( Qloh*X(18) - QCD*X(20) ) / Vloh
      XP(21) = ( QCD*X(20) - QU*X(21) ) / Vcd
      XP(22) = QU*( X(21) - X(22) ) / Vu
      XP(23) = QU*X(22)

      XP(24) = (QC*X(26) +QL*X(27)/NKpL +QB*X(28)/NKpB +(QK-QU)*X(42)
```

```
     1  + QH*X(30)/NKpH + QM*X(33)/NKpM + QA*X(34)/NKpA +QSk*X(35)/NKpSk
     1  + QBo*X(36)/NKpBo - QG*X(24))/VVE
        XP(25) = QG*(X(24)-X(25)/NKPG)/VG
        XP(26) = QG*(X(25)/NKPG-X(26))/VAR
        XP(27) = (FORM*CLintL*fup/KpL*X(4)*NMW/MW + QL*( X(26)-(
     1  QS*(X(26)-X(31)/NKPS)+ QI*(X(26)-X(32)/NKPI))/(QL) -X(27)/NKpL)
     1  -NCLintL*Nfup/NKpL*X(27) )/VL
        XP(28) = QB*(X(26)-X(28)/NKPB)/VB
        XP(29) = (Nfup*NPK*60*SK*Vptc* ( X(42) - X(29)/NKpK )
     1  + NCLrabs*X(41) - NCLintK*Nfup/11.7*X(29))/Vptc
        XP(30) = QH*(X(26)-X(30)/NKPH)/VH
        XP(31) = QS*(X(26)-X(31)/NKPS)/VS
        XP(32) = QI*(X(26)-X(32)/NKPI)/VI
        XP(33) = QM*(X(26)-X(33)/NKPM)/VM
        XP(34) = QA*(X(26)-X(34)/NKPA)/VA
        XP(35) = QSk*(X(26)-X(35)/NKPSk)/VSk
        XP(36) = QBo*(X(26)-X(36)/NKPBo)/VBo
        XP(37) = QK*(X(24)-X(37))/Vglm
        XP(38) = ( Nfup*GFR*X(37) - QS2*X(38) ) / VS1
        XP(39) = ( QS2*X(38) - QS3*X(39) ) / VS2
        XP(40) = ( QS3*X(39) - QS4*X(40) ) / VS3
        XP(41) = ( QS4*X(40) - Qloh*X(41) + NCLintK*Nfup/11.7*X(29)
     1  - NCLrabs*X(41) ) / VS4
        XP(42) = (( QK - Nfup*GFR )*X(37) - Nfup*NPK*60*SK*Vptc*
     1  ( X(42) - X(29)/NKpK ) - (QK-QU) * X(42) ) / Vrbl
        XP(43) = ( Qloh*X(41) - QCD*X(43) ) / Vloh
        XP(44) = ( QCD*X(43) - QU*X(44) ) / Vcd
        XP(45) = QU*( X(44) - X(45) ) / Vu
        XP(46) = QU*X(45)



C----------------------------------------------------------------------C
C----------------------------------------------------------------------C
C
        Return
        End
C######################################################################C
        Subroutine OUTPUT(Y,T,X)
        Implicit None
        Include 'globals.inc'
        Include 'model.inc'
```

```
      Real*8 Y(MaxNOE),T,X(MaxNDE)
      Real*8 Dose, MW, NMW
CC
C------------------------------------------------------------------C
C   Enter Output Equations Below    {e.g.   Y(1) = X(1)/P(2) }          C
C----c-------------------------------------------------------------C
      Dose = 2500
      MW=235.325
      NMW=277.362

      Y(1) = X(23)/Dose * 100
      Y(2) = X(46)/(Dose*NMW/MW)*100
      Y(3) = X(1)
      Y(4) = X(24)
C------------------------------------------------------------------C
C------------------------------------------------------------------C
C
      Return
      End
C####################################################################C
      Subroutine VARMOD(V,T,X,Y)
      Implicit None
      Include 'globals.inc'
      Include 'model.inc'
      Real*8 V(MaxNOE),T,X(MaxNDE),Y(MaxNOE), Sigma, Intercept
CC
C------------------------------------------------------------------C
C   Enter Variance Model Equations Below                              C
C          {e.g. V(1) = (PV(1) + PV(2)*Y(1))**2 }                   C
C----c-------------------------------------------------------------C
     Sigma = PV(1)
     Intercept = PV(2)
     V(1) = ( Intercept + Sigma*Y(1) ) ** 2
     V(2) = ( Intercept + Sigma*Y(2) ) ** 2
     V(3) = ( Intercept + Sigma*Y(3) ) ** 2
     V(4) = ( Intercept + Sigma*Y(4) ) ** 2

C------------------------------------------------------------------C
C------------------------------------------------------------------C
C
      Return
      End
```

```
C######################################################################C
      Subroutine COVMOD(Pmean, ICmean, PC)
C   Defines any covariate model equations (MLEM, ITS)
      Implicit None
      Include 'globals.inc'
      Include 'model.inc'
      Real*8 PC(MaxNCP)
      Real*8 Pmean(MaxNSP+MaxNDE), ICmean(MaxNDE)
CC
C----------------------------------------------------------------------C
C       Enter # of Covariate Parameters                                C
C----c-----------------------------------------------------------------C
      NCparam = 0       ! Enter # of Covariate Parameters.
CC
C----------------------------------------------------------------------C
C    Enter Symbol for Covariate Params {eg: PCsym(1)='CLRenal'}        C
C----c-----------------------------------------------------------------C
CC
C----------------------------------------------------------------------C
C    For the Model Params. that Depend on Covariates Enter the Equation C
C            {e.g. Pmean(1) =   PC(1)*R(2) }                           C
C----c-----------------------------------------------------------------C
C----------------------------------------------------------------------C
C----------------------------------------------------------------------C
C
      Return
      End
C######################################################################C
      Subroutine POPINIT(PmeanI,ICmeanI,PcovI,ICcovI, PCI)
C   Initial parameter values for population program parameters (ITS, MLEM)
      Implicit None
      Include 'globals.inc'
      Include 'model.inc'
      Integer I,J
      Real*8 PmeanI(MaxNSP+MaxNDE), ICmeanI(MaxNDE)
      Real*8 PcovI(MaxNSP+MaxNDE,MaxNSP+MaxNDE), ICcovI(MaxNDE,MaxNDE)
      Real*8 PCI(MaxNCP)
CC
C----------------------------------------------------------------------C
C   Enter Initial Values for Population Means                          C
C             {   e.g. PmeanI(1) = 10.0      }                         C
C----c-----------------------------------------------------------------C
```

```
CC
C-------------------------------------------------------------------C
C    Enter Initial Values for Pop. Covariance Matrix (Lower Triang.)      C
C            {   e.g. PcovI(2,1) = 0.25      }                            C
C----c--------------------------------------------------------------C
CC
C-------------------------------------------------------------------C
C    Enter Values for Covariate Model Parameters                          C
C            {   e.g. PCI(1) = 2.0      }                                 C
C----c--------------------------------------------------------------C
C-------------------------------------------------------------------C
C-------------------------------------------------------------------C
C
        Return
        End
C######################################################################C
        Subroutine PRIOR(Pmean,Pcov,ICmean,ICcov)
C   Parameter mean and covariance values for MAP estimation (ID,NPD,STS)
        Implicit None
        Include 'globals.inc'
        Include 'model.inc'
        Integer I,J
        Real*8 Pmean(MaxNSP+MaxNDE), ICmean(MaxNDE)
        Real*8 Pcov(MaxNSP+MaxNDE,MaxNSP+MaxNDE), ICcov(MaxNDE,MaxNDE)
CC
C-------------------------------------------------------------------C
C   Enter Nonzero Elements of Prior Mean Vector                           C
C             {   e.g. Pmean(1) = 10.0      }                             C
C----c--------------------------------------------------------------C
CC
C-------------------------------------------------------------------C
C    Enter Nonzero Elements of Covariance Matrix (Lower Triang.)          C
C            {   e.g. Pcov(2,1) = 0.25      }                             C
C----c--------------------------------------------------------------C
C-------------------------------------------------------------------C
C-------------------------------------------------------------------C
C
        Return
        End
C######################################################################C
        Subroutine SPARAM(PS,P,IC)
        Implicit None
```

```
      Include 'globals.inc'
      Real*8 PS(MaxNSECP), P(MaxNSP+MaxNDE), IC(MaxNDE), fdsum, fd1
CC
C-----------------------------------------------------------------------C
C    Enter Equations Defining Secondary Paramters                       C
C             {   e.g.   PS(1) = P(1)*P(2)    }                         C
C----c------------------------------------------------------------------C

C-----------------------------------------------------------------------C
C-----------------------------------------------------------------------C
C
      Return
      End

C#######################################################################C
      Subroutine AMAT(A)
      Implicit None
      Include 'globals.inc'
      Include 'model.inc'
      Integer I,J
      Real*8 A(MaxNDE,MaxNDE)
      DO I=1,Ndeqs
         Do J=1,Ndeqs
            A(I,J)=0.0D0
         End Do
      End Do
CC
C-----------------------------------------------------------------------C
C    Enter non zero elements of state matrix   {e.g.   A(1,1) = -P(1) }    C
C----c------------------------------------------------------------------C
C-----------------------------------------------------------------------C
C-----------------------------------------------------------------------C
C
      Return
      End
C#######################################################################C
```
